# Quality Control of the Traditional Patent Medicine Yimu Wan Based on SMRT Sequencing and DNA Barcoding

**DOI:** 10.3389/fpls.2017.00926

**Published:** 2017-05-31

**Authors:** Jing Jia, Zhichao Xu, Tianyi Xin, Linchun Shi, Jingyuan Song

**Affiliations:** Key Laboratory of Bioactive Substances and Resources Utilization of Chinese Herbal Medicine, Ministry of Education, Institute of Medicinal Plant Development, Peking Union Medical College and Chinese Academy of Medical SciencesBeijing, China

**Keywords:** quality control, single molecule real-time (SMRT) sequencing, DNA barcoding, circular-consensus sequencing (CCS), traditional patent medicine

## Abstract

Substandard traditional patent medicines may lead to global safety-related issues. Protecting consumers from the health risks associated with the integrity and authenticity of herbal preparations is of great concern. Of particular concern is quality control for traditional patent medicines. Here, we establish an effective approach for verifying the biological composition of traditional patent medicines based on single-molecule real-time (SMRT) sequencing and DNA barcoding. Yimu Wan (YMW), a classical herbal prescription recorded in the Chinese Pharmacopoeia, was chosen to test the method. Two reference YMW samples were used to establish a standard method for analysis, which was then applied to three different batches of commercial YMW samples. A total of 3703 and 4810 circular-consensus sequencing (CCS) reads from two reference and three commercial YMW samples were mapped to the ITS2 and *psbA-trnH* regions, respectively. Moreover, comparison of intraspecific genetic distances based on SMRT sequencing data with reference data from Sanger sequencing revealed an ITS2 and *psbA-trnH* intergenic spacer that exhibited high intraspecific divergence, with the sites of variation showing significant differences within species. Using the CCS strategy for SMRT sequencing analysis was adequate to guarantee the accuracy of identification. This study demonstrates the application of SMRT sequencing to detect the biological ingredients of herbal preparations. SMRT sequencing provides an affordable way to monitor the legality and safety of traditional patent medicines.

## Introduction

The sale of herbal supplement products was immediately halted at four major retailers in New York when DNA-based testing revealed that the products either could not be verified to contain the labeled substances or contained ingredients not listed on their labels (Schneiderman, 2015). This issue has increased global concern over the safety of herbal medicine and the quality control of traditional patent medicines. Traditional patent medicines are generally defined as plant-derived materials or preparations with therapeutic or other human health benefits that contain either raw or processed ingredients from one or more species (World Health Organization, 2000). The identifiable morphological and, often, the chemical features of the medicinal materials are altered when they are crushed and powdered, and misidentified herbal materials, adulterants, or biological contaminants are often included during the complex manufacturing process. Due to the lack of a thorough and standardized method for the assessment of herbal preparations, substandard and counterfeit drugs inevitably appear in the market and present potential public health problems. Thus, establishing an internationally recognized standard of quality control and evaluation for traditional patent medicines is crucial for industrialization and security applications worldwide (Cordell and Colvard, 2012).

DNA barcoding is a stand-alone authentication technique that is used to identify specimens and detect undescribed/cryptic species (Chen S. et al., 2014; Lo et al., 2015; Liu et al., 2016; Mishra et al., 2016). Since its introduction in 2003 (Hebert et al., 2003), this method has attracted considerable research interest and been extensively applied in different fields (Shen et al., 2013; Wu et al., 2015; Xin et al., 2015; Mishra et al., 2016). According to a study on the supervision of herbal products in North America, DNA tests have detected contamination and unlisted substitutions in the majority of products tested (Newmaster et al., 2013). A DNA-based identification test has been approved for incorporation into the Chinese Pharmacopoeia Committee (2015) and is recorded in the latest edition of the British Pharmacopoeia, appendix XI V (2016 edition) (The British Pharmacopoeia Commission, 2015). Despite the wide acceptance of these measures, they are insufficient for the quality control of mixed herbal preparations, which have been a focus of research. Thus, there is a critical need to implement broadly acceptable, powerful tools for detecting the mixed components of traditional patent medicines during industrial preparation. In the present study, we attempt to address these limitations using single-molecule real-time (SMRT) sequencing to determine the biological composition of a traditional herbal preparation. The SMRT sequencing platform is widely used in genome sequencing due to the long read lengths produced (Chen X. et al., 2014; Chaisson et al., 2015).

Yimu Wan (YMW), the research object of the present study, is a classical traditional Chinese herbal prescription originally developed in the ancient Chinese Qing Dynasty (17th century). Based on the Chinese Pharmacopoeia, the YMW preparation is composed of four types of herbal materials: Leonuri herba (*Leonurus japonicus* Houtt.), Angelicae sinensis radix [*Angelica sinensis* (Oliv.) Diels], Chuanxiong Rhizoma (*Ligusticum chuanxiong* Hort.) and Aucklandiae radix (*Aucklandia lappa* Decne.) (Supplementary Table S1). Among these materials, Leonuri herba, which has been used for thousands of years in China, exhibits the highest dosage and proportion in this preparation. Modern pharmacological studies have shown that the active components display numerous pharmacological actions, for example, showing effects on the uterus as well as cardioprotective, anti-oxidative, anti-cancer, analgesic, anti-inflammatory, neuroprotective and antibacterial properties (Shang et al., 2014). As recorded in the Chinese Pharmacopoeia, the methods applied for the evaluation of YMW include microscopic identification, thin-layer chromatography (TLC) identification and high-performance liquid chromatography (HPLC) determination, among others. However, there remain certain limitations of these methods for analyzing the biological ingredients of herbal preparations. For the successful identification of herbal ingredients and holistic quality control of these preparations, we suggest that SMRT sequencing be used in conjunction with DNA barcoding to analyze biological ingredients.

## Materials and Methods

### Authenticity of the Raw Materials

The four types of raw materials Leonuri herba (aerial part), Angelicae sinensis radix (root), Chuanxiong Rhizoma (rhizome), Aucklandiae radix (root) included in the composition of the reference YMW samples were purchased from a local drugstore. Additionally, *Panax ginseng* C. A. Meyer (root and rhizome) was used as a positive control (Song et al., 2012; Chen et al., 2013). To ensure the accuracy of the reference samples, all materials were identified via the DNA barcoding technique and Sanger sequencing (Supplementary Table S2), and morphological identification was performed according to the Chinese Pharmacopeia (Chinese Pharmacopoeia Committee, 2015).

### Standard Method of Quality Control for the Biological Composition of Herbal Preparations

#### YMW Reference Sample Production

Based on the methods described in the Chinese Pharmacopoeia, the reference YMW sample was formulated in the laboratory by the following procedures: (1) Four herbal materials, *Leonurus japonicas* (24 g), *Angelica sinensis* (12 g), *Ligusticum chuanxiong* (6 g), and *Aucklandia lappa* (2.25 g), in accordance with the proportions recorded in the Chinese Pharmacopoeia, were crushed into powder. (2) Ten grams of the mixed powder was weighed and marked as RF02. *P. ginseng* powder was added to a sample of the RF02 powder mixture at the same weight as *Aucklandia lappa*, which accounts for the lowest percentage of the prescription, to be used as a biological indicator for monitoring parameters. An additional experiment was conducted with another reference sample, RF01, the composition of which was the same as that of RF02 except that *P. ginseng* was omitted. (3) The powder was sieved and mixed uniformly. (4) The powder was mixed with double-distilled water and molded into pills.

#### DNA Extraction and Quantification

The DNA extraction procedure followed a previously published protocol for isolating total genomic DNA from plants according to the China Plant BOL Group (Li et al., 2011) and Chen et al. (2010) and Chen S. et al. (2014). Regarding the preparation of YMW, minor modifications to the method were made in the beginning steps. Prior to DNA extraction, 120 mg of the sample (RF02) was weighed and transferred into a 2 ml centrifuge tube. Extraction buffer (1 ml) consisting of 100 mM Tris-HCl (pH 8.0), 20 mM EDTA (pH 8.0), 0.7 M NaCl, 2% polyvinylpyrrolidone-40 (PVP-40) and 4‰ β-mercaptoethanol was added, followed by vortexing for 3 min and centrifugation for 3 min at 7500 rpm. The supernatant was removed, and the previous steps were repeated until the supernatant became almost clear. Total genomic DNA was isolated using the Plant Genomic DNA Kit [DP305, Tiangen Biotech (Beijing) Co., Ltd, China]. The incubation time for each sample was extended to 12 h, with gentle mixing at 56°C, after which 120 μl of sterile water was added to dissolve the DNA. The other steps were the same as outlined in the previously published protocol. The obtained DNA concentration was quantified on a NanoDrop 2000 spectrophotometer (Thermo Fisher Scientific, Inc., United States).

#### PCR Amplification and Purification

Two 5 bp tags were designed and added to the 5′ end of the universal ITS2 and *psbA-trnH* primers (Kress et al., 2005; Chen et al., 2010) to distinguish the sequences obtained from different regions. Different primers were used to the amplify ITS2 and *psbA-trnH* regions in the different samples. The designed primers used in the PCR for the detection of the different samples are listed in Supplementary Tables S3, S4. PCR amplifications were performed according to the DNA barcoding protocol recorded in the Chinese Pharmacopoeia and were carried out in an Applied Biosystems Veriti^TM^ Thermal Cycler (Thermo Fisher Scientific, Inc., United States). For each reaction, 1 μl MgCl_2_ (10 mM SBS Genetech Co., Ltd, China) and 2× Taq MasterMix (AidLab Biotechnologies Co., Ltd, China) were added to the PCR master mix. The PCR annealing temperature was increased to 58°C, and 40 cycles were run. A negative control reaction with no DNA template was included in the reactions. The DNA concentrations of the obtained amplicons were assessed via 2% agarose gel electrophoresis, and purification was performed with a QIAquick Gel Extraction kit (Qiagen). The DNA concentrations were measured on an Agilent 2100 bioanalyzer (Agilent Technologies, Inc., United States) with the Qubit platform (Thermo Fisher Scientific, Inc., United States).

#### Library Preparation and SMRT Sequencing

ITS2 and *psbA-trnH* amplicon sequencing was performed using the SMRT sequencing platform (PACBIO RSII, Pacific Biosciences of California, Inc., United States). The purified PCR amplicons were used to construct a SMRT sequencing library using the SMRTbell^TM^ Template Prep Kit 1.0 (part #100-259-100, Pacific Biosciences of California, Inc., United States)^1^. V2 primers were employed to bind the SMRTbell templates using the DNA/polymerase Binding Kit P6 v2 (part #100-372-700) and P6-DNA polymerase. The complexes were then bound to MagBeads (part #100-133-600) and transferred to a 96-well PCR plate for one round of SMRT cell sequencing using C4 reagents (part #100-356-200). The sequencing procedures were performed according to the manufacturer’s instructions and a previous study (Li et al., 2014).

#### Data Processing and Biological Composition Analysis

**FIGURE 1:**
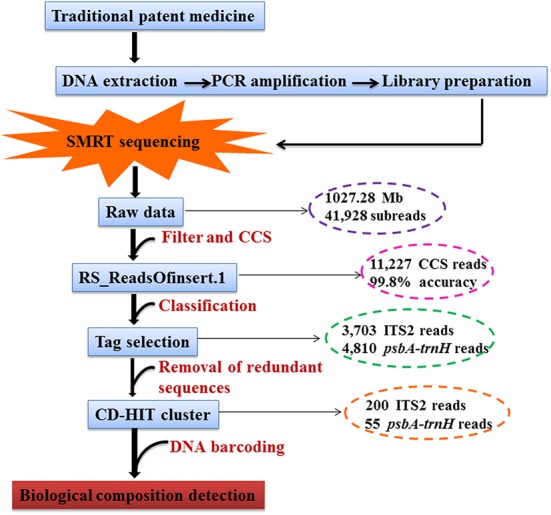
Standard quality control method for traditional patent medicines.

We used the SMRT Analysis Server 2.3.0 (Pacific Biosciences of California, Inc., United States) provided by PacBio followed by RS_ReadsOfInsert to obtain the circular-consensus sequencing (CCS) sub-read dataset, which was run through these programs to filter out the standard sequences (minimum read length of insert, 250 bp; maximum read length of insert, 600 bp; minimum full passes, 10; minimum prediction accuracy, 99%). Based on the tags, the CCS reads from RF02 were separated, and data libraries were constructed using Perl scripts. Next, the CCS reads from each library were clustered, and redundant sequences were removed using CD-HIT software^2^. The clustered reads were then identified in the DNA Barcoding System for Identifying Herbal Medicine^3^ using the Basic Local Alignment Search Tool (BLAST). The powerful interactive species analysis tool MEGAN6 was used to cluster the BLAST files for generating the phylogenetic tree. The SMRT sequencing pipeline of the data analysis is shown in **Figure 1**. Proofreading and contig assembly of the sequencing peak diagrams of all CCS reads were performed using CodonCode Aligner 6.0.2 (CodonCode Co., United States). The ITS2 region was obtained using the HMMer annotation method based on the Hidden Markov model (HMM) to remove the 5.8S and 28S sections at both ends of the sequences. Based on the sequence lengths of the raw materials obtained via Sanger sequencing, the CCS reads obtained via SMRT sequencing with sequence lengths of more than 10 bases were removed. The screened reads were then used for the calculation of genetic distances. The genetic distances were computed with the Kimura 2-Parameter (K2P) model.

### Validation of the Standard Method

In accordance with the quality control method for herbal preparations established using RF02, an additional procedure was conducted with another reference sample, RF01. The composition of RF01 was the same as that of RF02 except that it did not include *P. ginseng*. To distinguish between PCR amplicons derived from different samples, two additional 5 bp tags were designed and added to the 5′ end of the universal primers of the ITS2 and *psbA-trnH* sequences (Supplementary Tables S3, S4). All steps of the operation were consistent with the standard method to verify its replicability.

### Application of the Standard Method to the Commercial YMW Herbal Preparation

Three batches of commercial YMW (namely, YMW01, Lot No.: 3015152; YMW02, Lot No.: 2015395; and YMW03, Lot No.: 2015616) from the same manufacturer were randomly purchased from different drug stores. These specimens were sampled and tested using the same method applied to the reference sample RF02. Honey is employed as the adhesive in the commercial YMW herbal preparation and was removed with extraction buffer before DNA extraction. As a result, the commercial samples weighed much more than the reference sample, at 300 mg per 2.0 ml centrifuge tube. The remaining steps were performed according to the standard method.

## Results

### Sanger Sequencing of the Four Raw Materials Constituting YMW

**FIGURE 2:**
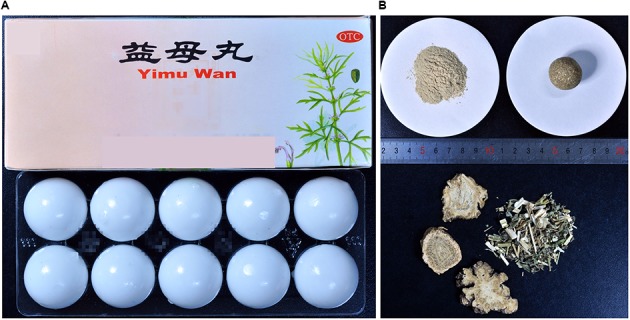
Photographs of the Yimu Wan (YMW) samples that were genetically audited in this study using single-molecule real-time (SMRT) sequencing. **(A)** Commercial YMW samples randomly purchased from drug stores. **(B)** Raw materials and preparation of the reference YMW sample.

The four raw materials constituting YMW were authenticated via DNA barcoding and morphological identification to ensure the accuracy of the analysis of the biological ingredients of the reference samples. The ITS2 and *psbA-trnH* regions were successfully sequenced via standard Sanger sequencing. All obtained sequences were submitted to the DNA Barcoding System for Identifying Herbal Medicine^4^, and species determination was based on the best hit and the smallest genetic distance of the query sequence obtained through BLAST analysis. The results indicated that the four raw materials (**Figure 2**), *Leonurus japonicus*, *Ligusticum chuanxiong*, *Angelica sinensis*, and *Aucklandia lappa*, all yielded ITS2 and *psbA-trnH* sequences. Additionally, the results for the species in the reference samples were consistent with the records in the Chinese Pharmacopeia. All of the ITS2 and *psbA-trnH* sequence haplotypes of each species were submitted to GenBank and are listed in Supplementary Table S5. From the above results, we concluded that ITS2 and *psbA-trnH* can be used as biomarkers for the identification of plant ingredients and to verify the accuracy of reference samples.

### Establishment of A Quality Control Method for Traditional Patent Medicines via SMRT Sequencing

In our study, two reference samples (RF01-02) and three commercial samples (YMW01-03) of YMW were used as the study materials, and the data were output through a process of DNA extraction, PCR amplification and purification and SMRT sequencing. We have provided a detailed protocol for the targeted capture of the ITS2 and *psbA-trnH* gene panel, and the protocol for the DNA library preparation, barcoding, purification and quantification steps is described in detail (**Figure 1**).

**FIGURE 3:**
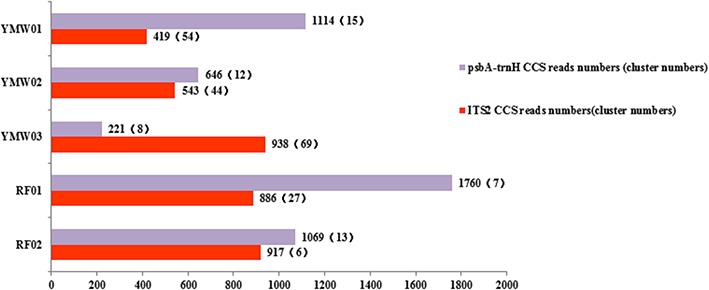
Analysis of the SMRT sequencing data, number of clusters and number of circular-consensus sequencing (CCS) reads.

From the total output of 1027.28 Mb, 41,928 reads were obtained via SMRT sequencing. After the data quality control procedure, the SMRT sequencer generated 11,227 trimmed and filtered CCS reads for all samples (**Figure 3**). A total of 2714 CCS reads were unmatched as redundant sequences and were removed based on tag selection. Finally, 3703 and 4810 CCS reads were mapped to the ITS2 and *psbA-trnH* regions, which clustered into 200 and 55 sequence clusters, respectively (Supplementary Table S6). As a specific species, we chose *Leonurus japonicus* of commercial YMW01 as the object of study. In YMW01, 419 and 1114 CCS reads were matched as ITS2 and *psbA-trnH* regions, which clustered into 54 and 15 sequence clusters, respectively. A total of 95 and 999 CCS reads of *Leonurus japonicus* were mapped to the ITS2 and *psbA-trnH* regions, which clustered into 6 and 5 sequence clusters, respectively. Sequencing information for each species is summarized in Supplementary Tables S6, S7.

### Analysis of the Biological Ingredients of the Reference and Commercial YMW Samples

In total, we obtained 1803 ITS2 and 2829 *psbA-trnH* sequence CCS reads for the two reference samples (RF01 and RF02). Based on the ITS2 sequences, four prescribed herbal materials, *Leonurus japonicas*, *Ligusticum chuanxiong*, *Angelica sinensis*, and *Aucklandia lappa*, were detected in each of the reference samples. In addition, the positive control, *P. ginseng*, was successfully detected in the RF02 reference sample. Additionally, the *psbA-trnH* sequences of *Leonurus japonicas*, *Ligusticum chuanxiong*, and *Angelica sinensis* were detected in each of the reference samples. *Aucklandia lappa* and *P. ginseng* were not detected in the two reference samples. In general, with the combination of the two DNA barcodes, four prescribed species and the positive control species (*P. ginseng*) were identified, confirming the feasibility of the untargeted method for biological ingredient analysis of traditional patent medicines (**Table 1**).

**Table 1:** single-molecule real-time (SMRT) sequencing of species detected in the Yimu Wan (YMW) samples based on the ITS2 and *psbA-trnH* regions.

Species	YMW01		YMW02		YMW03		RF01		RF02	
	ITS2	*psbA-trnH*	ITS2	*psbA-trnH*	ITS2	*psbA-trnH*	ITS2	*psbA-trnH*	ITS2	*psbA-trnH*
*Leonurus japonicas*	√	√	√	√	√	√	√	√	√	√
*Ligusticum Chuanxiong*	√	√	√	√	√	√	√	√	√	√
*Angelica sinensis*	√	√	√	√	√	√	√	√	√	√
*Aucklandia lappa*			√				√		√	
*Panax ginseng*									√	

An in-depth analysis of the species identified in three commercial YMW samples was performed using the same method employed for the reference specimens. In total, 1900 ITS2 and 1981 *psbA-trnH* sequence CCS reads were obtained from the three commercial samples (YMW01-03). Based on the ITS2 sequences analyzed, three prescribed raw materials, *Leonurus japonicas*, *Ligusticum chuanxiong*, and *Angelica sinensis*, were detected in the three commercial samples, whereas *Aucklandia lappa* was not found in YMW01 and YMW03 but was detected in YMW02. Regarding the *psbA-trnH* sequences, with the exception of *Aucklandia lappa*, the prescribed raw materials were all detected in the three commercial samples. It is possible that the genomic DNA of *Aucklandia lappa* was too degraded to be amplified.

### Adulterant and Contaminant Detection

The SMRT sequencing results showed that contaminant species were present in the reference samples, although all of the raw materials were verified. For instance, *Angelica amurensis*, an adulterant of *Angelica sinensis*, was detected in RF01. When we authenticated the samples, we chose one small piece at random; thus, adulterants were not detected. Furthermore, the *Artemisia* genus was detected in both RF01 and RF02, and species from the families Convolvulaceae, Asteraceae, Fabaceae, Lamiaceae, Apiaceae, Amaranthaceae, Apocynaceae, Ranunculaceae, and Aristolochiaceae were detected in RF01 (Supplementary Table S7).

In addition to the prescribed species, many non-listed plant species were detected in the commercial samples and were presumed to be contaminants (Supplementary Table S7). Regarding the contaminant species, the CCS reads that mapped to the ITS2 and *psbA-trnH* regions were classified into 28 families including 47 genera (for ITS2) and 6 families including 8 genera (for *psbA-trnH*). The five most common plant genera were *Angelica, Humulus, Ipomoea, Artemisia*, and *Amaranthus* based on the combined ITS2 and *psbA-trnH* results. We used MEGAN6 to cluster the BLAST files to analyze the (**Supplementary Figures S1**, **S2**) phylogeny and the relative abundance of species detected in the YMW samples from different batches.

### Profiles of the Intraspecific and Interspecific Diversity of Different Prescribed Species Based on SMRT Sequencing Analysis

As recorded in the Chinese Pharmacopoeia, the YMW preparation is composed of four herbal materials: *Leonurus japonicus*, *Angelica sinensis*, *Ligusticum chuanxiong*, and *Aucklandia lappa*. We estimated intraspecific and interspecific genetic distances using the K2P model for the different prescribed species detected in the two reference and three commercial YMW samples (**Tables 2**–**5**). Based on the obtained intraspecific distances from the Sanger sequencing and SMRT sequencing, the ITS2 and *psbA-trnH* intergenic spacer was found to exhibit high intraspecific divergence. Comparison of the SMRT-based sequences and the Sanger-based sequences revealed many variable sites. The results also showed that in the sequences obtained through SMRT sequencing compared with Sanger sequencing within a species, there was evidence of sites of variation, which was significantly random. At the species level, *Leonurus japonicas* proportionally accounted for the largest dosage in the YMW preparation and was selected for analyzing the sites of variation. We chose *Leonurus japonicus*, which had the lowest cluster number in the RF02 reference sample, as the object of study (**Supplementary Figure S3**). The results showed that although the cluster number of *Leonurus japonicus* was lowest, the variable sites changed randomly. We also compared the interspecific genetic distances of the prescribed species with the interspecific genetic distances within genera based on the ITS2 and *psbA-trnH* regions. The result also showed that the minimum interspecific genetic distances of the ITS2 and the *psbA-trnH* intergenic spacer were much higher than the corresponding maximum intraspecific genetic distances. Use of the ITS2 and *psbA-trnH* intergenic spacer as a DNA barcode was suitable for the identification of four types of herbal materials in YMW samples and related species. Because the genus *Aucklandia* was represented by only one species (*Aucklandia lappa*) in this study, *Aucklandia lappa* and related species were not analyzed in depth^5^. All the sequences are available on GenBank (Supplementary Table S5).

**Table 2:** Intraspecific genetic distances determined via Sanger sequencing vs. SMRT sequencing for the prescribed species in the YMW samples based on the ITS2 region.

Sample ID	YMW01	YMW02	YMW03	RF01	RF02
*Leonurus japonicas*	0.0085 ± 0.0164	0.0314 ± 0.0462	0.0048 ± 0.0086	0.0083 ± 0.0109	0.0030 ± 0.0044
*Ligusticum chuanxiong*	0.0126 ± 0.0101	0.0129 ± 0.0100	0.0178 ± 0.0187	0.0121 ± 0.0130	0.0270 ± 0.0126
*Angelica sinensis*	0.0117 ± 0.0181	0.0055 ± 0.0110	0.0124 ± 0.0157	0.0016 ± 0.0034	0.0025 ± 0.0035
*Aucklandia lappa*	-	0	-	0	0.0068 ± 0.0032

*The symbol “-” indicates no CCS reads. “0” indicates that the number of CCS reads was ≤2, preventing calculation of intraspecific distance.*

**Table 3:** Intraspecific genetic distances determined via Sanger sequencing and SMRT sequencing for the prescribed species in the YMW samples based on the *psbA-trnH* region.

Sample ID	YMW01	YMW02	YMW03	RF01	RF02
*Leonurus japonicas*	0.0023 ± 0.0031	0.0035 ± 0.0043	0.0021 ± 0.0029	0.0022 ± 0.0028	0.0020 ± 0.0025
*Ligusticum chuanxiong*	0.0075 ± 0.0073	0.0050 ± 0.0093	0.0041 ± 0.0055	0	0.0215 ± 0.0186
*Angelica sinensis*	0.0032 ± 0.0060	0.0025 ± 0.0032	0.0078	0.0011 ± 0.0022	0.0012 ± 0.0030
*Aucklandia lappa*	-	-	-	-	-

*The symbol “-” indicates no CCS reads. “0” indicates that the number of CCS reads was ≤2, preventing calculation of intraspecific distance.*

**Table 4:** Interspecific genetic distances determined via species of the same genus vs. SMRT sequencing for the prescribed species in the YMW samples based on the ITS2 region.

Sample ID	YMW01	YMW02	YMW03	RF01	RF02
*Leonurus japonicas*	0.1224 ± 0.0100	0.1436 ± 0.0374	0.1209 ± 0.0086	0.1250 ± 0.0087	0.1180 ± 0.0065
*Ligusticum chuanxiong*	0.0557 ± 0.0063	0.0557 ± 0.0069	0.0574 ± 0.0099	0.0563 ± 0.0067	0.0509 ± 0.0071
*Angelica sinensis*	0.0835 ± 0.0187	0.0906 ± 0.0108	0.0881 ± 0.0062	0.0897 ± 0.0048	0.0935 ± 0.0045
*Aucklandia lappa*	-	-	-	-	-

**Table 5:** Interspecific genetic distances determined via species of the same genus and SMRT sequencing for the prescribed species in the YMW samples based on the *psbA-trnH* region.

Sample ID	YMW01	YMW02	YMW03	RF01	RF02
*Leonurus japonicas*	0.0349 ± 0.0037	0.0363 ± 0.0044	0.0347 ± 0.0035	0.0351 ± 0.0034	0.0346 ± 0.0030
*Ligusticum chuanxiong*	0.0543 ± 0.0149	0.0499 ± 0.0071	0.0493 ± 0.0082	0.0282	0.0644 ± 0.0471
*Angelica sinensis*	0.0166 ± 0.0043	0.0154 ± 0.0045	0.0198	0.0141 ± 0.0043	0.0146 ± 0.0051
*Aucklandia lappa*	-	-	-	-	-

## Discussion

### An Effective Approach for the Quality Control of Traditional Patent Medicines

A SMRT sequencing strategy was established in this study to evaluate the quality of traditional patent medicines. SMRT sequencing appears to be an effective method for monitoring the biological composition of traditional patent medicines. This technique has not been applied previously to sequencing single or mixed herbal materials. Here, we developed a CCS strategy involving SMRT DNA sequencing technology that guarantees the accuracy of identification (**Figure 1**). Due to the diverse properties of herbal plants and medicinal components, the different pre-processing procedures used for medicinal materials, and the various production processes for herbal preparations, all of the tested samples were washed in extraction buffer to ensure DNA quality. Successful DNA isolation from herbal preparations is the key to successful sequencing.

In our study, all four of the prescribed herbal materials in the RF02 reference sample were detected using ITS2 and *psbA-trnH* sequences. *P. ginseng*, which was specifically added to RF02 as a positive control for co-amplification with the target sequences, was also detected. These results indicated the SMRT sequencing approach is reliable for detecting all of the prescribed species in herbal preparations. Furthermore, the validation experiment with RF01 produced results consistent with those for RF02, which confirmed the flexibility of the SMRT sequencing strategy for monitoring the biological composition of traditional patent medicines. For the analysis of commercial samples, three different batches from the same manufacturer were subjected to the same methods used for the reference samples. Based on the combination of the ITS2 and *psbA-trnH* sequences, three, four, and three prescribed species were detected in YMW01, YMW02, and YMW03, respectively (**Table 1**). These results indicate that the method established in this study can be applied to commercial samples.

Before the formal evaluations, the Sanger sequencing for mixed species was evaluated. As it could not be successfully applied to the mixed species, it was used for a single species only. Sanger sequencing information based on ITS2 was successfully obtained when applied to the four species in YMW individually, as shown in **Supplementary Figure S4**, but not when applied to the mixed species, as shown in **Supplementary Figure S5**. Fortunately, SMRT sequencing could identify all the species in the mixed herbal materials. Sequencing DNA molecules based on the real-time detection of processive DNA polymerization at a base-pair resolution has long been proposed. SMRT sequencing has been applied to the full-length transcriptome of *Salvia miltiorrhiza* Bunge (Xu et al., 2015), to the *de novo* assembly and detection of the chloroplast genomes of *Fritillaria* species (Li et al., 2014), to the sequencing of the desiccation-tolerant grass *Oropetium thomaeum* (VanBuren et al., 2015) and to the potato late blight-resistance gene (Witek et al., 2016). The present study demonstrated the application of SMRT sequencing as a high-throughput alternative for the analysis of multiple targets in herbal preparations and showed that it can be applied to the quality control of traditional patent medicines.

### Advantages and Disadvantages of SMRT Sequencing for Identifying Traditional Patent Medicines

This study aimed to assess the applicability of SMRT sequencing and DNA barcoding to the verification of the biological composition of traditional patent medicines and aimed to establish a new method. SMRT sequencing has been demonstrated to be a powerful, universal approach for the highly accurate *de novo* assembly and sensitive SNP detection of chloroplast genomes (Li et al., 2014). In addition, second-generation DNA sequencing technology has been applied to analyze the biological ingredients of Chinese patent medicines. For example, a study of Traditional Chinese Medicines (TCMs) seized by Australia border protection officials was conducted using the Roche GS Junior system (Coghlan et al., 2012), and a study of the biological ingredients of Liuwei Dihuang Wan was performed based on 454 GS FLX Titanium sequencing (Cheng et al., 2014). The Roche 454 system was widely used because of the long read lengths it provided; however, Roche has discontinued its 454 sequencing business^6^. The Illumina HiSeq 2000 features the larger output, but the short read length remains a major shortcoming. Moreover, a complete run spans 3–10 days. Compared with second generation sequencers, PacBio RS has several advantages. First, the sample preparation is very rapid; it takes 4–6 h instead of days. In addition, a sequencing reaction on a single SMRT Cell requires only 90 min, and the data are output in real time, providing more flexibility than second-generation sequencing. Second, the turnover rate is rapid; runs are finished within a day. Third, the average read length is 1300 bp, which is longer than that of any second-generation sequencing technology (Liu et al., 2012). The SMRT sequencing technique analyzes single DNA molecules and produces extraordinarily long reads with high consensus accuracy, which is not currently possible with other next-generation sequencing-based methods. A unique feature of SMRT sequencing is the ability to detect DNA base modifications directly and automatically by measuring the rate of nucleotide incorporation during sequencing (Flusberg et al., 2010; Clark et al., 2012; Schadt et al., 2013). Extending the read length enhances the *de novo* assembly of genomes, the characterization of genomic structural variations (Grad et al., 2012; Koren et al., 2013), and the analysis of targeted sequencing regions (Carneiro et al., 2012; Smith et al., 2012).

In the present study, the strategy of using the CCS method (with multiple reading of the template DNA by DNA polymerase), which is typical in the PacBio SMRT sequencing process, greatly reduced the error rate for the raw reads (Travers et al., 2010; Li et al., 2014). Accordingly, all of the obtained sequences were high-quality, single-molecule reads that exceeded 10 kb in length. A greater number of fragment passes also helps to improve the accuracy of CCS reads; with appropriate filtering, CCS reads can reach an accuracy of >99% (Fichot and Norman, 2013). In addition, the improved P6-C4 chemistry was adopted to ensure sequencing accuracy. Furthermore, the CCS reads from each library were clustered, and redundant sequences were removed with CD-HIT software, which ensured the stability of the quality value (QV). The QV of each base call was significantly increased in the CCS sub-reads, allowing highly confident copy detection at a low variant frequency to guarantee identification accuracy. Moreover, using SMRT sequencing can greatly shorten the time required for detecting and analyzing samples. These considerations also apply to other sequencing platforms, such as SEQUEL. The basic principle of SEQUEL is the same as that of PacBio RSII, and the sequencing yield is approximately seven times that of PacBio RSII. The application of the higher yield SEQUEL sequencing platform to biological components of Chinese patent medicines will improve the sensitivity of this approach^7^.

The intraspecific genetic distances of the different prescribed species in the YMW samples were calculated in this study. As shown in **Tables 2**, **3**, the intraspecific distances based on the ITS2 and *psbA-trnH* regions presented some differences within a single species among different samples. For example, the intraspecific distances based on the ITS2 sequences of *Leonurus japonicus* in the two reference and three commercial YMW samples ranged from 0.0030 (RF02) to 0.0314 (YMW02). A possible explanation for this result is that the sequences obtained through SMRT sequencing contained some errors. Fortunately, these errors did not affect the efficacy of species identification. As the ITS2 region is present in multiple copies in the genome, there are potentially dozens of different sequences in a single sample (Song et al., 2012). Moreover, PCR bias and artifact formation might have occurred in the multitemplate PCR assay and provided incorrect information on the abundance and diversity of genes.

Single-molecule real-time sequencing has several potential limitations. First, relative to the cost associated with the 454 pyrosequencing and Illumina MiSeq sequencing platforms, SMRT sequencing instruments are expensive (Quail et al., 2012). Second, to achieve reliable sequencing of the data, the experimental environment must be clear. Additionally, SMRT sequencing cannot be effectively performed in the presence of DNA degradation this arises from the processing of traditional patent medicines.

### Comparison of Identification Results between the Reference and Commercial YMW Samples

In this study, SMRT sequencing combined with DNA barcoding successfully detected both the prescribed herbal materials and the positive control in the reference samples, even given the low percentages of *P. ginseng* and *Aucklandia lappa* in the mixtures. The approach presented here can be considered a holistic quality control strategy for monitoring the biological composition of traditional patent medicines. Moreover, based on the ITS2 and *psbA-trnH* sequences, *Aucklandia lappa* could be detected via Sanger sequencing and was found in both of the reference samples but only one of the commercial samples (YMW02). A possible reason for the different identification profiles of the processed herbal materials might be that the genomic DNA was damaged. Another reason might be that *Aucklandia lappa* was not added to the herbal preparations during processing. Because *Aucklandia lappa* grows in high mountain regions with a cool climate, it can be difficult to obtain. The results showed that the quality of the genomic DNA was the most important factor influencing detection success. The *Artemisia* genus was detected in both RF01 and RF02, and species from the families Convolvulaceae, Asteraceae, Fabaceae, Lamiaceae, Apiaceae, Amaranthaceae, Apocynaceae, Ranunculaceae, and Aristolochiaceae were detected in RF01 (Supplementary Table S7). It is possible that contaminants were introduced during the extraction of total DNA from the preparations; plant powders might have become airborne or been carried over during the experimental process. The SMRT sequencing analysis suggested that possible cross-contamination can be monitored at any stage during the manufacturing, handling or laboratory analysis of the plant material. The results of SMRT sequencing showed that 28 plant families (based on ITS2 and *psbA-trnH* sequences combined) were detectable in the commercial samples, reflecting the powerful identification ability of the method. Possible explanations for this contamination can be classified as follows: (1) identification errors or the introduction of pollutants during raw material collection; (2) sharing of the production line during the processing procedure; (3) intentional addition to the herbal preparations, as observed for *Leonurus sibiricus*, the adulterant of *Leonurus japonicas*; and (4) unintentional mixing into the herbal preparations, as observed for the species belonging to *Ipomoea, Rhododendron*, and *Amaranthus*. These species are used in many other herbal preparations and can easily cause cross-contamination.

Moreover, we found contamination in commercial samples from plants exhibiting known toxicity, side effects and/or negative interactions with other herbs, supplements, or medications. For example, we found that the genus *Humulus* was present in all of the commercial samples. Members of this genus contain toxic compounds that may pose serious health risks (Bent, 2008). Thus, our detection of this genus provides an example of a contaminant that has known toxic compounds and pharmacological activity among the species in the preparation. The results indicate that the method developed in this study enables the reliable detection of source species and could be utilized by manufacturers for quality assurance of raw materials, contamination control during production, and final preparation.

### Prospects for the Future Application of the Quality Control Method for Traditional Patent Medicines

The authentication of traditional patent medicines is challenging; although the application of SMRT sequencing to analyze herbal medicines has not been previously attempted, it is likely to become the best practical method for genetically auditing the species composition of multiple traditional patent medicines. Although there is considerable evidence of the health benefits of herbal preparations (Wolsko et al., 2005; Nikam et al., 2012), the industry suffers from unethical activities by certain manufacturers; these activities include false advertising, product substitution, contamination and the use of fillers. Although many manufacturers provide products with consistent levels of active ingredients through a process known as chemical standardization, this technique has uncertain effects on the safety and efficacy of the final preparations (Raynor et al., 2011). Many dangers of commercial herbal preparations have been revealed by DNA technology-based studies, which have identified the contamination of herbal preparations with poisonous plants. Hence, evaluation of the biological composition of traditional patent medicines is essential.

Given the increasing popularity and global demand for natural remedies and medicines, ensuring a safe and sustainable supply of quality products is paramount. Ayurvedic medicine practiced in India, Kampo medicine in Japan, Traditional Chinese Medicine (TCM), and Unani medicine in the Middle East and South Asia continue to apply herbal medicine as an alternative. The use of PacBio SMRT sequencing coupled with DNA barcoding is of interest to the traditional patent medicines industry because it permits the effective monitoring of quality and safety during production. Future advances that allow the analysis of nucleic acid sequences from damaged or fragmented template DNA at a large scale will have great potential for use in the identification of traditional patent medicines.

## Conclusion

We have provided an effective approach for monitoring the biological composition of traditional patent medicines based on SMRT sequencing and DNA barcoding. Using the CCS strategy for SMRT sequencing analysis was adequate to guarantee the accuracy of identification. This study demonstrates the application of SMRT sequencing to detect the biological ingredients in herbal preparations. This approach represents an affordable way to monitor the legality and safety of traditional patent medicines. In the near future, this method may become a powerful tool for the quality control of traditional patent medicines, and it shows strong potential for application.

## Author Contributions

JJ, JS, and ZX designed the study. JJ performed experiments. JJ, ZX, TX, and LS analyzed the data. JJ, JS, and ZX drafted the manuscript. All authors have read and approved the final manuscript.

## Conflict of Interest Statement

The authors declare that the research was conducted in the absence of any commercial or financial relationships that could be construed as a potential conflict of interest.

## Footnotes

1 http://www.pacificbiosciences.com/

2 http://weizhongli-lab.org/cd-hit/

3 http://www.tcmbarcode.cn/en/

4 http://www.tcmbarcode.cn

5 http://www.efloras.org

6 http://www.natap.org/2013/newsUpdates/101513_02.htm

7 http://www.pacb.com/products-and-services/pacbio-systems/sequel/

## Supplementary material

FIGURE S1 Phylogeny and relative abundance of species detected in YMW from three different batches based on ITS2.

FIGURE S2 Phylogeny and relative abundance of species detected in YMW from three different batches based on *psbA-trnH*.

FIGURE S3 Analysis of sites of variation in *Leonurus japonicus* in a reference YMW sample (RF02).

FIGURE S4 Sanger sequencing information based on ITS2 of four single species in YMW.

FIGURE S5 Sanger sequencing results based on ITS2 of mixed species in YMW.
