# Supplementary figures and images for: Quality Control of the Traditional Patent Medicine Yimu Wan Based on SMRT Sequencing and DNA Barcoding
Source: Front Plant Sci. 2017 May 31;8:926. doi: 10.3389/fpls.2017.00926 (PMC5449480; doi:10.3389/fpls.2017.00926)

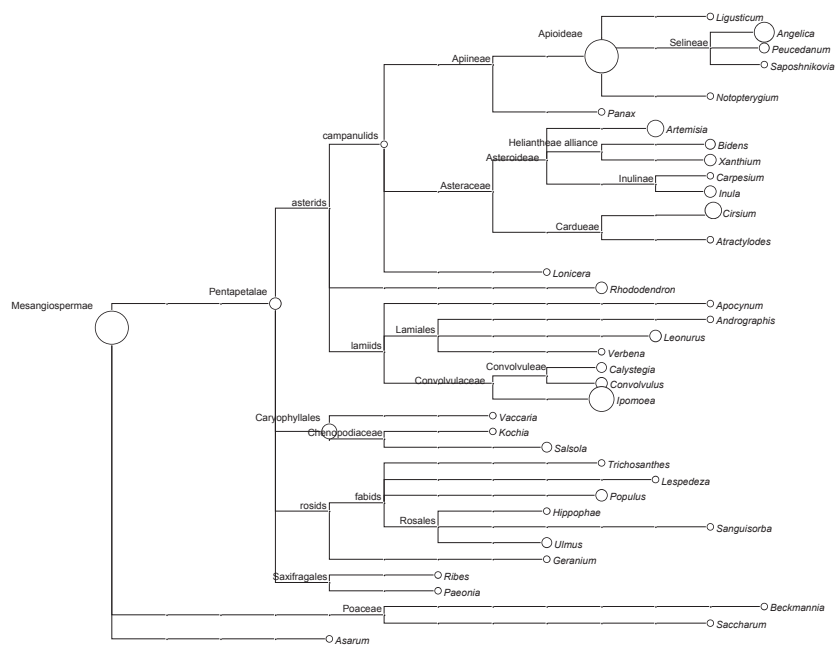

Supplement: FIGURE S1 — Phylogeny and relative abundance of species detected in YMW from three different batches based on ITS2. [file Image_1.PDF]

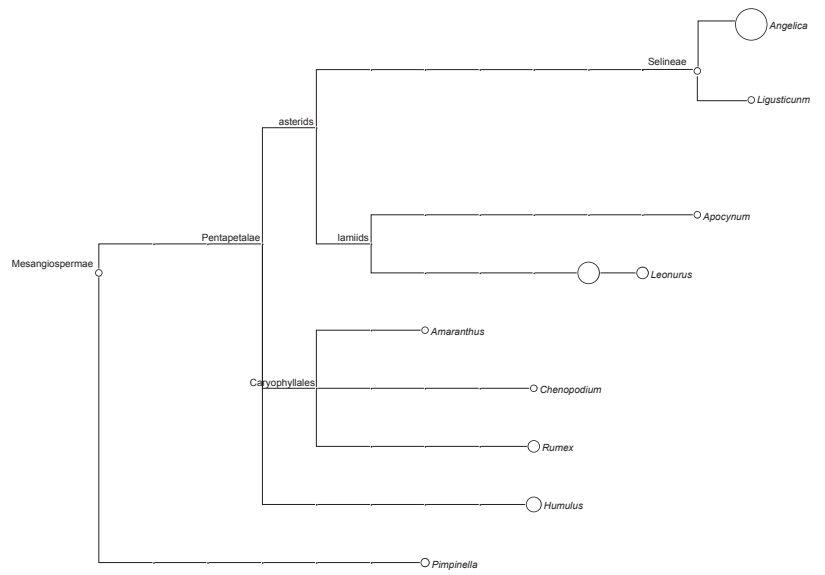

Supplement: FIGURE S2 — Phylogeny and relative abundance of species detected in YMW from three different batches based on psbA-trnH. [file Image_2.PDF]

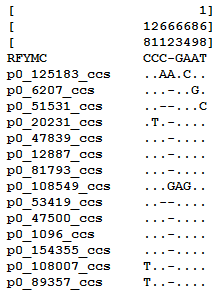

Supplement: FIGURE S3 — Analysis of sites of variation in Leonurus japonicus in a reference YMW sample (RF02). [file Image_3.TIF]

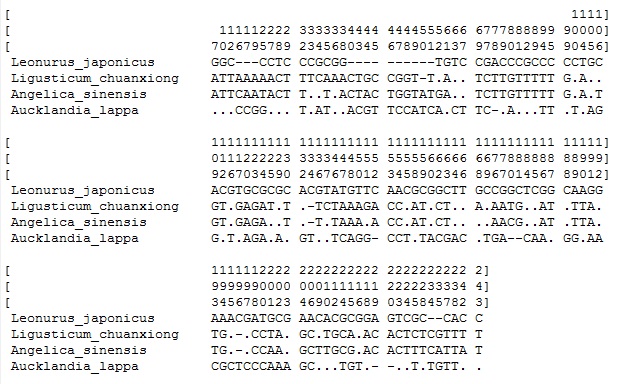

Supplement: FIGURE S4 — Sanger sequencing information based on ITS2 of four single species in YMW. [file Image_4.JPEG]

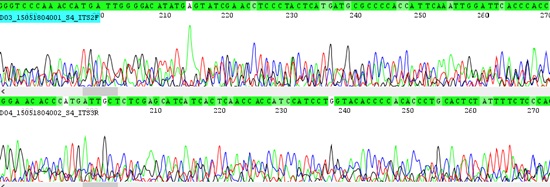

Supplement: FIGURE S5 — Sanger sequencing results based on ITS2 of mixed species in YMW. [file Image_5.JPEG]
