# Supplementary material for: Quality Control of the Traditional Patent Medicine Yimu Wan Based on SMRT Sequencing and DNA Barcoding
Source: Front Plant Sci. 2017 May 31;8:926. doi: 10.3389/fpls.2017.00926 (PMC5449480; doi:10.3389/fpls.2017.00926)
Supplement: Supplementary file 6 [file Table_1.DOCX]

**Table S1. Prescription composition of YMW in the Chinese Pharmacopoeia.**

| **Species** | **Latin name** | **Chinese name** | **Family** | **Genus** |
| --- | --- | --- | --- | --- |
| Leonuri herba | *Leonurus japonicus* Houtt. | Yimucao | Lamiaceae | *Leonurus* |
| Chuanxiong Rhizoma | *Ligusticum chuanxiong* Hort. | Chuanxiong | Apiaceae | *Ligusticum* |
| Angelicae sinensis radix | *Angelica sinensis* (Oliv.) Diels | Danggui | Apiaceae | *Angelica* |
| Aucklandiae radix | *Aucklandia lappa* Decne. | Muxiang | Asteraceae | *Aucklandia* |
