# Supplementary material for: Quality Control of the Traditional Patent Medicine Yimu Wan Based on SMRT Sequencing and DNA Barcoding
Source: Front Plant Sci. 2017 May 31;8:926. doi: 10.3389/fpls.2017.00926 (PMC5449480; doi:10.3389/fpls.2017.00926)
Supplement: Supplementary file 7 [file Table_2.DOCX]

**Table S2. ITS2 and *psbA-trnH* gene primer sequences for Sanger sequencing**

| Sequence name | Primer name | Primer sequence(5'→3') | Annealing  temp. (℃) | Primer  reference |
| --- | --- | --- | --- | --- |
| ITS2 | S2F | ATGCGATACTTGGTGTGAAT | 56℃ | [1] |
|  | S3R | GACGCTTCTCCAGACTACAAT |  |  |
| *psbA-trnH* | fwd PA | GTTATGCATGAACGTAATGCTC | 55℃ | [2] |
|  | rev TH | CGCGCATGGTGGATTCACAATCC |  |  |

[1] Chen, S.L., Yao, H., Han, J.P., Liu, C., Song, J.Y., Shi, L.C., et al. (2010). Validation of the ITS2 region as a novel DNA barcode for identifying medicinal plant species. *PLoS One.*5, e8613. doi: 10.1371/journal.pone.0008613

[2] Kress W.J., Wurdack K.J., Zimmer E.A., Weigt L.A., Janzen D.H. (2005). Use of DNA barcodes to identify flowering plants. *Proc Natl Acad Sci U S A*,102:8369–74. doi:  [10.1073/pnas.0503123102](https://dx.doi.org/10.1073%2Fpnas.0503123102)
