# Supplementary material for: Quality Control of the Traditional Patent Medicine Yimu Wan Based on SMRT Sequencing and DNA Barcoding
Source: Front Plant Sci. 2017 May 31;8:926. doi: 10.3389/fpls.2017.00926 (PMC5449480; doi:10.3389/fpls.2017.00926)
Supplement: Supplementary file 8 [file Table_3.DOCX]

**Table S3. ITS2 primers used for SMRT sequencing.**

| Sample ID | ITS2 forward primer sequence (5'-3') | ITS2 reverse primer sequence(5'-3') | 5-bp tags |
| --- | --- | --- | --- |
| YMW01 | TATAGATGCGATACTTGGTGTGAAT | TATAGGACGCTTCTCCAGACTACAAT | TATAG |
| YMW02 | AAGTCATGCGATACTTGGTGTGAAT | AAGTCGACGCTTCTCCAGACTACAAT | AAGTC |
| YMW03 | CCTAGATGCGATACTTGGTGTGAAT | CCTAGGACGCTTCTCCAGACTACAAT | CCTAG |
| RF01 | GGAATATGCGATACTTGGTGTGAAT | GGAATGACGCTTCTCCAGACTACAAT | GGAAT |
| RF02 | ACACTATGCGATACTTGGTGTGAAT | ACACTGACGCTTCTCCAGACTACAAT | ACACT |
