# Supplementary material for: Quality Control of the Traditional Patent Medicine Yimu Wan Based on SMRT Sequencing and DNA Barcoding
Source: Front Plant Sci. 2017 May 31;8:926. doi: 10.3389/fpls.2017.00926 (PMC5449480; doi:10.3389/fpls.2017.00926)
Supplement: Supplementary file 9 [file Table_4.DOCX]

**Table S4. *psbA-trnH* primers used for SMRT sequencing.**

| Sample ID | *psbA-trnH* forward primer sequence (5'-3') | *psbA-trnH* reverse primer sequence(5'-3') | 5-bp tags |
| --- | --- | --- | --- |
| YMW01 | GTATGGTTATGCATGAACGTAATGCTC | GTATGCGCGCATGGTGGATTCACAATCC | GTATG |
| YMW02 | TGAATGTTATGCATGAACGTAATGCTC | TGAATCGCGCATGGTGGATTCACAATCC | TGAAT |
| YMW03 | TAATCGTTATGCATGAACGTAATGCTC | CGCGCATGGTGGATTCACAATCC | — |
| RF01 | AAGTCGTTATGCATGAACGTAATGCTC | AAGTCCGCGCATGGTGGATTCACAATCC | AAGTC |
| RF02 | GCGTAGTTATGCATGAACGTAATGCTC | GCGTACGCGCATGGTGGATTCACAATCC | GCGTA |
