# Supplementary material for: Quality Control of the Traditional Patent Medicine Yimu Wan Based on SMRT Sequencing and DNA Barcoding
Source: Front Plant Sci. 2017 May 31;8:926. doi: 10.3389/fpls.2017.00926 (PMC5449480; doi:10.3389/fpls.2017.00926)
Supplement: Supplementary file 10 [file Table_5.DOCX]

**Table S5. Sequence information deposited in GenBank**

| **Latin name** | **GenBank No. of ITS2** | **GenBank No. of *psbA-trnH*** |
| --- | --- | --- |
| *Leonurus japonicus* | KX675126 | EU590862 |
| *Ligusticum chuanxiong* | GQ434693 | GQ435306 |
| *Angelica sinensis* | GQ434694 | KJ999580 |
| *Aucklandia lappa* | KX674793 | KM233920 |
| *Panax ginseng* | KX674876 | GQ435398 |
| *Leonurus cardiaca* | KT695255 | FJ513116 |
| *Angelica tianmuensis* | DQ270194 | KC887960 |
| *Ligusticum sinense* | KC295051 | KC295131 |
