# Supplementary material for: Quality Control of the Traditional Patent Medicine Yimu Wan Based on SMRT Sequencing and DNA Barcoding
Source: Front Plant Sci. 2017 May 31;8:926. doi: 10.3389/fpls.2017.00926 (PMC5449480; doi:10.3389/fpls.2017.00926)
Supplement: Supplementary file 11 [file Table_6.DOCX]

**Table S6. Data analysis of cluster numbers (CCS read numbers) from the reference and commercial YMW samples via SMRT**

**sequencing.**

| **Species** | **YMW01** | | **YMW02** | | **YMW03** | | **RF01** | | **RF02** | |
| --- | --- | --- | --- | --- | --- | --- | --- | --- | --- | --- |
|  | **ITS2** | ***psbA-trnH*** | **ITS2** | ***psbA-trnH*** | **ITS2** | ***psbA-trnH*** | **ITS2** | ***psbA-trnH*** | **ITS2** | ***psbA-trnH*** |
| *Leonurus japonicus* | 6(95) | 5(999) | 3(6) | 2(160) | 8(125) | 2(167) | 3(88) | 4(402) | 1(14) | 2(214) |
| *Ligusticum Chuanxiong* | 2(58) | 2(9) | 3(59) | 3(81) | 7(209) | 1(45) | 2(14) | 1(1) | 1(12) | 3(3) |
| *Angelica sinensis* | 2(17) | 2(95) | 2(277) | 2(393) | 1(9) | 1(1) | 3(728) | 2(1357) | 1(883) | 8(852) |
| *Aucklandia lappa* |  |  | 1(1) |  |  |  | 1(1) |  | 1(2) |  |
| *Panax ginseng* |  |  |  |  |  |  |  |  | 1(1) |  |
