# Supplementary material for: Quality Control of the Traditional Patent Medicine Yimu Wan Based on SMRT Sequencing and DNA Barcoding
Source: Front Plant Sci. 2017 May 31;8:926. doi: 10.3389/fpls.2017.00926 (PMC5449480; doi:10.3389/fpls.2017.00926)
Supplement: Supplementary file 12 [file Table_7.DOCX]

**Table S7.** **CCS read numbers of the adulterant and contaminant species detected in 5 YMW samples with SMRT sequencing based on the ITS2 and *psbA-trnH* regions.**

| **NO.** | **Family** | **Genus** | **YMW01** | | **YMW02** | | **YMW03** | | **RF01** | | **RF02** | |
| --- | --- | --- | --- | --- | --- | --- | --- | --- | --- | --- | --- | --- |
|  |  |  | **ITS2** | ***psbA-trnH*** | **ITS2** | ***psbA-trnH*** | **ITS2** | ***psbA-trnH*** | **ITS2** | ***psbA-trnH*** | **ITS2** | ***psbA-trnH*** |
| 1 | Convolvulaceae | *Convolvulus* | 2 |  |  |  | 1 |  | 1 |  |  |  |
|  |  | *Ipomoea* | 15 |  | 26 |  | 43 |  | 3 |  |  |  |
|  |  | *Calystegia* | 12 |  |  |  |  |  |  |  |  |  |
| 2 | Cannabaceae | *Cannabis* | 3 |  |  |  |  |  |  |  |  |  |
|  |  | *Humulus* | 117 | 3 | 39 | 36 | 76 | 1 |  |  |  |  |
| 3 | Asteraceae | *Taraxacum* | 1 |  |  |  |  |  |  |  |  |  |
|  |  | *Lactuca* | 6 |  |  |  |  |  |  |  |  |  |
|  |  | *Actites* | 5 |  | 1 |  | 5 |  |  |  |  |  |
|  |  | *Carpesium* | 1 |  |  |  |  |  |  |  |  |  |
|  |  | *Cirsium* | 2 |  | 2 |  | 5 |  |  |  |  |  |
|  |  | *Atractylodes* |  |  | 2 |  | 1 |  |  |  |  |  |
|  |  | *Xanthium* |  |  | 2 |  | 7 |  |  |  |  |  |
|  |  | *Inula* |  |  | 3 |  | 9 |  | 1 |  |  |  |
|  |  | *Bidens* |  |  |  |  | 3 |  | 1 |  |  |  |
|  |  | *Artemisia* | 6 |  | 15 |  | 36 |  | 3 |  | 5 |  |
| 4 | Geraniaceae | *Geranium* | 2 |  |  |  | 1 |  |  |  |  |  |
| 5 | Malvaceae | *Abutilon* | 6 |  | 2 |  | 4 |  |  |  |  |  |
| 6 | Ulmaceae | *Ulmus* | 5 |  |  |  | 2 |  |  |  |  |  |
| 7 | Verbenaceae | *Verbena* | 1 |  |  |  |  |  |  |  |  |  |
| 8 | Scandiceae | *Daucus* | 1 |  |  |  |  |  |  |  |  |  |
| 9 | Fabaceae | *Glycine* | 1 |  |  |  | 4 |  |  |  |  |  |
|  |  | *Astragalus* |  |  | 1 |  |  |  |  |  |  |  |
|  |  | *Vigna* |  |  |  |  | 2 |  |  |  |  |  |
|  |  | *Lespedeza* |  |  |  |  | 1 |  |  |  |  |  |
|  |  | *Glycyrrhiza* |  |  |  |  |  |  | 13 |  |  |  |
| 10 | Ericaceae | *Robinia* | 2 |  |  |  | 1 |  |  |  |  |  |
| 11 | Lamiaceae | *Rhododendron* | 2 |  | 1 |  | 2 |  | 1 |  |  |  |
| 12 | Apiaceae | *Perilla* |  |  |  |  | 1 |  |  |  |  |  |
|  |  | *Angelica* | 52 |  | 84 | 6 | 365 | 3 | 3 |  |  |  |
|  |  | *Peucedanum* |  |  | 6 |  | 10 |  |  |  |  |  |
|  |  | *Tetrataenium* |  |  |  | 1 |  |  |  |  |  |  |
|  |  | *Pimpinella* |  | 1 |  |  |  |  |  |  |  |  |
|  |  | *Saposhnikovia* |  |  |  |  |  |  | 12 |  |  |  |
|  |  | *Ligusticum* |  |  |  |  |  |  |  |  |  |  |
|  |  | *Notopterygium* |  |  |  |  |  |  | 6 |  |  |  |
| 13 | Poaceae | *Saccharum* | 1 |  |  |  |  |  |  |  |  |  |
|  |  | *Beckmannia* | 1 |  |  |  |  |  |  |  |  |  |
| 14 | Amaranthaceae | *Amaranthus* | 1 | 2 | 1 |  | 10 |  | 2 |  |  |  |
| 15 | Salicaceae | *Populus* | 1 |  |  |  | 2 |  |  |  |  |  |
| 16 | Paeoniaceae | *Paeonia* | 1 |  |  |  |  |  |  |  |  |  |
| 17 | Caryophyllaceae | *Vaccaria* | 1 |  |  |  |  |  |  |  |  |  |
| 18 | Brassicaceae | *Lepidium* | 1 |  |  |  |  |  |  |  |  |  |
| 19 | Caprifoliaceae | *Lonicera* |  |  | 1 |  |  |  |  |  |  |  |
| 20 | Apocynaceae | *Apocynum* |  | 1 |  |  |  |  | 1 |  |  |  |
| 21 | Paniceae | *Neurachne* |  |  | 1 |  |  |  |  |  |  |  |
| 22 | Cucurbitaceae | *Trichosanthes* |  |  | 5 |  |  |  |  |  |  |  |
| 23 | Chenopodiaceae | *Chenopodium* |  |  |  |  |  | 1 |  |  |  |  |
|  |  | *Kochia* |  |  |  |  | 2 |  |  |  |  |  |
|  |  | *Salsola* |  |  | 1 |  | 1 |  |  |  |  |  |
| 24 | Elaeagnaceae | *Hippophae* |  |  | 1 |  |  |  |  |  |  |  |
| 25 | Acanthaceae | *Andrographis* |  |  | 3 |  |  |  |  |  |  |  |
| 26 | [Polygonaceae](http://www.ncbi.nlm.nih.gov/Taxonomy/Browser/wwwtax.cgi?mode=Undef&id=3615&lvl=3&keep=1&srchmode=1&unlock) | *Rumex* |  | 4 |  | 4 |  | 3 |  |  |  |  |
|  |  | *Rheum* |  |  | 1 |  |  |  |  |  |  |  |
| 27 | Ranunculaceae | *Aconitum* |  |  | 1 |  |  |  |  |  |  |  |
|  |  | *Prunus* |  |  |  |  |  |  | 1 |  |  |  |
| 28 | Rosaceae | *Sanguisorba* |  |  | 1 |  |  |  |  |  |  |  |
| 29 | [Grossulariaceae](https://www.ncbi.nlm.nih.gov/Taxonomy/Browser/wwwtax.cgi?mode=Undef&id=23066&lvl=3&keep=1&srchmode=1&unlock) | *Ribes* |  |  |  |  | 1 |  |  |  |  |  |
| 30 | Aristolochiaceae | *Asarum* |  |  |  |  |  |  | 7 |  |  |  |
| **31 Total** | | | **249** | **11** | **200** | **12** | **595** | **8** | **55** |  | **5** |  |
